# The Apolipoprotein B/A1 Ratio is Associated With Metabolic Syndrome Components, Insulin Resistance, Androgen Hormones, and Liver Enzymes in Women With Polycystic Ovary Syndrome

**DOI:** 10.3389/fendo.2021.773781

**Published:** 2022-01-05

**Authors:** Hui He, Jiaxing Feng, Shike Zhang, Yu Wang, Jian Li, Jingshu Gao, Jing Cong, Yi Gong, Xiaoke Wu

**Affiliations:** ^1^ Heilongjiang University of Chinese Medicine, Harbin, China; ^2^ Department of Rehabilitation Medicine, Southern University of Science and Technology Yantian Hospital, Shenzhen, China; ^3^ Department of Obstetrics and Gynecology, First Affiliated Hospital of Heilongjiang University of Chinese Medicine, Harbin, China; ^4^ Department of Obstetrics and Gynecology, First Affiliated Hospital, Guizhou Medical University, Guiyang, China; ^5^ Beilun District People’s Hospital, The First Affiliated College of Medicine, Beilun Branch of Zhejiang University, Ningbo, China; ^6^ Heilongjiang Province Hospital, Harbin, China

**Keywords:** polycystic ovary syndrome, metabolic syndrome, insulin resistance, hyperandrogenism, liver enzyme, apolipoprotein ratio

## Abstract

**Aim:**

To evaluate the association between the apolipoprotein B/A1 ratio (ApoB/ApoA1) and metabolic and endocrine parameters in women with polycystic ovary syndrome (PCOS).

**Methods:**

This study was a secondary analysis of the Acupuncture and Clomiphene for Chinese Women with Polycystic Ovary Syndrome trial (PCOSAct), and 957 subjects with available ApoB and ApoA1 measurements were included. Tests for linear trends and linear regression were used to assess the relation between the ApoB/ApoA1 ratio and metabolic and endocrine parameters. Logistic regression was used to estimate the association between the ratio and risk of metabolic syndrome (MetS) and insulin resistance (IR). The receiver operating characteristics (ROC) curve was used to determine the predictive value of the ApoB/ApoA1 ratio for MetS and IR.

**Results:**

The results showed that the ApoB/ApoA1 ratio was positively associated with waist circumference, systolic blood pressure, total cholesterol, triglycerides, low-density lipoprotein, fasting plasma glucose, fasting insulin, homeostatic model assessment-insulin resistance, high free testosterone, high free androgen index, alanine transferase, aspartate transferase, and higher prevalence of MetS and IR, but was negatively correlated with high-density lipoprotein and sex hormone-binding globulin after adjusting for age and body mass index. Logistic regression showed that compared with the ApoB/ApoA1 ratio in first quartile, those in the fourth quartile demonstrated a higher risk of MetS (OR: 24.48, 95%CI: 8.54–70.15, P trend <0.001) and IR (OR: 1.78, 95%CI: 1.10–2.87, P trend <0.05) after adjusting for confounding factors. ROC curve results showed that the AUC_MetS_ was 0.84 (95%CI: 0.81–0.86) and had 86.8% sensitivity and 70.3% specificity with a threshold value of 0.64, and the AUC_IR_ was 0.68 (95%CI: 0.64–0.71) and had 74.3% sensitivity and 58.2% specificity with a threshold value of 0.56.

**Conclusions:**

Increased ApoB/ApoA1 ratio was associated with worse MetS components, IR, and elevated androgen hormones and liver enzymes. The ratio might be a useful tool to screen for MetS and IR in PCOS patients.

## Introduction

Polycystic ovary syndrome (PCOS) is the most common endocrine disorder in reproductive-age women (1), generally presenting as oligo-/amenorrhea, hyperandrogenism (HA), and infertility (2). In addition, PCOS patients often suffer from metabolic abnormalities, namely, overweight or obesity (3), insulin resistance (IR) (4), dyslipidemia (5), non-alcoholic fatty liver disease (NAFLD) (6), metabolic syndrome (MetS) (7), and cardiovascular diseases (CVD) in the long-term (8). IR, which is the impairment of insulin action, plays an intrinsic role in the pathogenesis of PCOS and aggravates the reproductive and metabolic disorders in patients with PCOS (9). It is therefore important to identify a tool to screen for metabolic and endocrine parameters in women with PCOS.

Apolipoproteins are liver-produced proteins that are responsible for lipid transportation and redistribution. Among them, apolipoprotein A1 (ApoA1), a constituent of high-density lipoprotein (HDL), is involved in the process of reverse transport of peripheral cholesterol to the liver and thus has an anti-atherogenic effect (10). Apolipoprotein B (ApoB), which consists of chylomicrons, intermediate-density lipoprotein, very-low-density lipoprotein (VLDL), and low-density lipoprotein (LDL), is responsible for transporting cholesterol to peripheral cells and is a representative example of atherogenic lipoproteins (11). Therefore, the ApoB/ApoA1 ratio might reflect the balance between atherogenic lipoproteins and anti-atherogenic lipoproteins (12). Numerous studies have shown that the ApoB/ApoA1 ratio is strongly associated with MetS (13–16) and might be an independent predictor of IR (17) among different ethnicities. Moreover, the ApoB/ApoA1 ratio is a better predictor of lipoprotein-related risk of cardiovascular disease (CVD) than traditional lipid indexes (18–20), and ApoB and ApoA1 are correlated with creatine kinase (CK) in myocardial infarction (21). Meanwhile, several studies have indicated that the ratio is related to NAFLD (22, 23) and to liver function markers such as alanine transferase (ALT) (24, 25).

To the best of our knowledge, only two published studies have analyzed the association of the ApoB/ApoA1 ratio with endocrine and metabolic characteristics in adults and adolescents with PCOS, respectively (26, 27). Their studies concluded that the ApoB/ApoA1 ratio is strongly connected with IR and MetS, but their results indicated that the mechanisms of PCOS might be different between adolescents and adults. Thus, the relation between ApoB/ApoA1 and the metabolic characteristics in PCOS remains inconclusive.

The aim of this study was to evaluate the associations between the ApoB/ApoA1 ratio and metabolic and endocrine profiles, namely, MetS, IR, androgen hormones, and cardiac and liver enzymes. We also investigated whether the ApoB/ApoA1 ratio could be used as an indicator to predict MetS and IR.

## Material and Methods

### Participants

This study was a cross-sectional secondary analysis of the Acupuncture and Clomiphene for Chinese Women with Polycystic Ovary Syndrome Trial (PCOSAct), which was conducted between 2012 and 2015 in mainland China. The clinical trial was registered at chictr.org.cn (ChiCTR-TRC-12002081) and Clinical Trials.gov (NCT01573858). The protocol was approved by all ethics committees at the local study sites. The study protocol and primary manuscript have been published elsewhere (28, 29). A total of 1,000 infertile women with PCOS were recruited in the trial, and the diagnosis of PCOS was based on the modified Rotterdam criteria, namely chronic oligomenorrhea or amenorrhea together with clinical/biochemical hyperandrogenemia and/or polycystic ovarian morphology confirmed by transvaginal ultrasound. The details of the inclusion and exclusion criteria are described in the protocol (28).

### Anthropometric Measurements

All participants underwent a physical examination at the baseline visit: age, height, weight, waist circumference (WC), systolic blood pressure (SBP), and diastolic blood pressure (DBP). Body mass index (BMI) was calculated as weight divided by height squared.

### Biochemical Measurements

All blood samples at the baseline visit were collected on the third day of the menstrual cycle after a 12-hour overnight fast and were analyzed at the core laboratory of the Heilongjiang University of Chinese Medicine. Biochemical measurements included total cholesterol (TC), triglycerides (TG), LDL, HDL, ApoA1, ApoB, fasting plasma glucose (FPG), fasting insulin (FIN), total testosterone (TT), free testosterone (FT), sex hormone-binding globulin (SHBG), CK, creatine kinase isoenzyme MB (CKMB), lactate dehydrogenase (LDH), ALT, and aspartate transferase (AST). TG and TC were measured by the N-(3-sulfopropyl)-3-methoxy-5-methylaniline method (Wako Diagnostics). HDL and LDL were measured by direct-method assays. ApoA1 and ApoB levels were measured by the polyethylene glycol-enhanced immunoturbidimetric assay (Maker, Chengdu, China). FPG was measured by hexokinase assay (Maker, Chengdu, China). FIN was measured by electro-chemiluminescence immune assay (ECLIA) (Roche Diagnostic, Basel, Switzerland). TT and SHBG were analyzed by chemiluminescence immunoassay (Siemens Diagnostic, Munich, Germany). FT was measured by radioimmunoassay. CK, LDH, ALT, and AST were determined by the IFCC method. CKMB was analyzed by the selective inhibition method. The free androgen index (FAI) was calculated by the formula: FAI = TT (nmol/L)/SHBG (nmol/L) × 100. The homeostasis model assessment of insulin resistance (HOMA-IR) index was calculated by the equation: HOMA-IR = FIN (mIU/ml) × FBG (mmol/L)/22.5 (30). Insulin resistance (IR) status was considered as a HOMA-IR ≥2.69 (31).

### Metabolic Syndrome

MetS was defined by presenting three or more of the following five items (1): WC >88 cm; (2) SBP ≥130 mmHg or DBP ≥85 mmHg; (3) FPG level of 110–126 mg/dl (to convert to millimoles per liter, multiply by 0.0555); (4) TG level ≥150 mg/dl (to convert to millimoles per liter, multiply by 0.0113); and (5) HDL level <50 mg/dl (to convert to millimoles per liter, multiply by 0.0259) (1).

### Statistical Analysis

SPSS Statistics (IBM SPSS, Inc., Chicago, IL, USA version 26.0) was used for data analyses. Continuous variables are presented as means ± standard deviations, and categorical variables are presented as frequencies and percentages. Anthropometric and biochemical parameters and the prevalence of MetS and IR in the participants across the ApoB/ApoA1 quartiles were compared using tests for linear trends. Linear regression was used to determine the correlations between the ApoB/ApoA1 ratio and the characteristics of the study population. In addition, multivariable logistic regression analysis was used to calculate the odds ratio (OR) with 95% confidence interval (CI) for the associations between the ApoB/ApoA1 ratio (the independent variable) and MetS and IR status (the dependent variables). Finally, receiver operating characteristic (ROC) curves were used to evaluate the predictive value of the ApoB/ApoA1 ratio for MetS and IR. The area under the curve (AUC) was measured, and the optimal cut-off values of the ApoB/ApoA1 ratio were also calculated by the highest Youden index (sensitivity + specificity − 1) (32). A P‐value <0.05 was considered to be statistically significant.

## Results

A total of 957 PCOS patients with available ApoB and ApoA1 measurements were included in the analysis. The ApoB/ApoA1 ratio was calculated and classified into four quartiles (Q1: ≤0.45, n = 240; Q2: 0.46–0.60, n = 239; Q3: 0.61–0.74, n = 239; and Q4 >0.74, n = 239). Among them, 190 women were diagnosed with MetS and 401 women were diagnosed with IR.

### The Anthropometric and Biochemical Characteristics of the Participants Across the Quartiles of ApoB/ApoA1 Ratio

The anthropometric and biochemical characteristics across quartiles of ApoB/ApoA1 ratio are summarized in **Table 1**. Rising trends were observed for age, BMI, WC, SBP, DBP, TC, TG, LDL, FPG, FIN, HOMA-IR, FT, FAI, LDH, ALT, and AST across the ApoB/ApoA1 ratio quartiles (P-trend <0.01 for all), while declining trends were seen for HDL, SHBG, and CKMB across the four groups (P-trend <0.05 for all). The prevalence of MetS in each category of increasing ApoB/ApoA1 ratio in PCOS patients was 1.7, 5.9, 22.6, and 49.4%, respectively. The corresponding prevalence of IR was 22.92, 33.05, 49.79, and 61.92%, respectively. The differences observed across the quartiles of ApoB/ApoA1 ratio for the prevalence of MetS and IR were all statistically significant (P-trend <0.001).

**Table 1:** Comprehensive metabolic parameters of the included PCOS participants according to quartile of ApoB/ApoA1 ratio.

Variables	Quartile of ApoB/ApoA1 ratio				*P*-_trend_
	Q1 (≤0.45)	Q2 (0.46–0.60)	Q3 (0.61–0.74)	Q4 (>0.74)	
n	240	239	239	239	
Age (year)	27.25 ± 3.00	27.75 ± 3.18	28.10 ± 3.48	28.51 ± 3.38	<0.001
BMI (kg/m^2^)	21.57 ± 3.18	23.20 ± 3.92	25.69 ± 4.18	26.35 ± 3.86	<0.001
WC (cm)	79.28 ± 9.60	82.36 ± 10.40	89.26 ± 11.08	90.84 ± 10.64	<0.001
SBP (mmHg)	109.08 ± 9.42	111.92 ± 9.84	112.81 ± 8.70	115.46 ± 8.39	<0.001
DBP (mmHg)	72.73 ± 7.66	74.84 ± 8.19	75.35 ± 7.58	76.42 ± 7.41	<0.001
TC (mmol/L)	4.17 ± 0.85	4.48 ± 0.91	4.85 ± 0.92	5.45 ± 1.19	<0.001
TG (mmol/L)	1.03 ± 0.55	1.28 ± 0.66	1.76 ± 0.86	2.21 ± 1.00	<0.001
LDL (mmol/L)	2.32 ± 0.55	2.76 ± 0.64	3.13 ± 0.70	3.67 ± 0.94	<0.001
HDL (mmol/L)	1.55 ± 0.38	1.32 ± 0.32	1.16 ± 0.27	1.06 ± 0.30	<0.001
APOA1 (g/L)	1.68 ± 0.32	1.53 ± 0.31	1.46 ± 0.28	1.36 ± 0.26	<0.001
APOB (g/L)	0.63 ± 0.13	0.80 ± 0.17	0.97 ± 0.19	1.20 ± 0.26	<0.001
ApoB/ApoA1	0.38 ± 0.05	0.52 ± 0.04	0.66 ± 0.04	0.89 ± 0.13	<0.001
FPG (mmol/L)	4.88 ± 0.89	5.00 ± 0.78	5.03 ± 0.89	5.24 ± 1.20	<0.001
FIN (pmol/L)	67.85 ± 69.32	83.08 ± 77.32	108.48 ± 96.31	120.01 ± 81.21	<0.001
HOMA-IR	2.26 ± 2.84	2.72 ± 2.73	3.70 ± 4.06	4.27 ± 3.89	<0.001
TT (nmol/L)	1.67 ± 0.66	1.62 ± 0.61	1.63 ± 0.66	1.75 ± 0.64	0.20
FT (pg/ml)	2.13 ± 0.89	2.25 ± 0.81	2.29 ± 0.88	2.51 ± 0.74	<0.001
FAI	3.97 ± 3.10	4.94 ± 3.57	6.39 ± 4.40	8.10 ± 5.07	<0.001
SHBG (nmol/L)	60.37 ± 36.93	46.88 ± 30.72	34.67 ± 20.96	27.93 ± 16.16	<0.001
CK	57.37 ± 35.83	56.39 ± 42.01	59.01 ± 36.22	58.60 ± 30.26	0.547
CKMB	3.51 ± 6.57	2.50 ± 2.35	1.89 ± 2.03	2.58 ± 4.81	0.019
LDH	81.65 ± 41.04	82.77 ± 39.62	83.18 ± 51.41	92.90 ± 53.60	0.001
ALT (U/L)	6.75 ± 7.63	7.31 ± 7.59	7.46 ± 4.94	10.82 ± 8.34	<0.001
AST (U/L)	12.23 ± 6.94	11.61 ± 4.71	11.72 ± 4.98	13.72 ± 6.33	<0.001
MetS, n (%)	4 (1.7%)	14 (5.9%)	54 (22.6%)	118 (49.4%)	<0.001
IR, n (%)	55 (22.92%)	79 (33.05%)	119 (49.79%)	148 (61.92%)	<0.001

BMI, body mass index; WC, waist circumference; SBP, systolic blood pressure; DBP, diastolic blood pressure; TC, total cholesterol; TG, triglycerides; LDL, low-density lipoprotein; HDL, high-density lipoprotein; APOA1, apolipoprotein A1; APOB, apolipoprotein B; FPG, fasting plasma glucose; FIN, fasting insulin; HOMA-IR, homeostatic model assessment-insulin resistance; TT, total testosterone; FT, free testosterone; FAI, free androgen index; SHBG, sex hormone-binding globulin; CK, creatine kinase; CKMB, creatine kinase MB; LDH, lactate dehydrogenase; ALT, alanine transferase; AST, aspartate transferase; MetS, metabolic syndrome; IR, insulin resistance.

### Linear Regression Analysis Between the ApoB/ApoA1 Ratio and the Anthropometric and Biochemical Characteristics of the PCOS Patients

There was a significant positive correlation between the ApoB/ApoA1 ratio and age, BMI, WC, SBP, DBP, TC, TG, LDL, FPG, FIN, HOMA-IR, TT, FT, FAI, LDH, ALT, and AST (P <0.01 for all), while there was a significant inverse relationship between the ApoB/ApoA1 ratio and SHBG and HDL (P <0.001 for both). After adjusting for age and BMI, the association between the ApoB/ApoA1 ratio and DBP, TT, and LDH disappeared (**Table 2**).

**Table 2:** Linear associations between the ApoB/ApoA1 ratio and metabolic parameters.

Variables	Coefficient β	*P*-value	Coefficient β adjusted for age and BMI	*P*-value
WC (cm)	0.415	<0.001	0.055	0.007
SBP (mmHg)	0.239	<0.001	0.114	0.001
DBP (mmHg)	0.161	<0.001	0.067	0.06
TC (mmol/L)	0.479	<0.001	0.493	<0.001
TG (mmol/L)	0.510	<0.001	0.449	<0.001
LDL (mmol/L)	0.601	<0.001	0.627	<0.001
HDL (mmol/L)	-0.502	<0.001	-0.477	<0.001
APOA1 (g/L)	-0.380	<0.001	-0.400	<0.001
APOB (g/L)	0.788	<0.001	0.771	<0.001
Lipoprotein (mg/L)	0.134	<0.001	0.155	<0.001
FPG (mmol/L)	0.158	<0.001	0.081	0.022
FIN (pmol/L)	0.251	<0.001	0.091	0.006
HOMA-IR	0.235	<0.001	0.089	0.009
TT (nmol/L)	0.069	0.034	0.063	0.085
FT (pg/ml)	0.179	<0.001	0.122	0.001
FAI	0.374	<0.001	0.262	<0.001
SHBG (nmol/L)	−0.391	<0.001	−0.288	<0.001
CK (U/L)	0.015	0.658	−0.045	0.223
CKMB (U/L)	−0.062	0.155	−0.037	0.457
LDH (U/L)	0.085	0.01	0.038	0.296
ALT (U/L)	0.210	<0.001	0.110	0.002
AST (U/L)	0.152	<0.001	0.077	0.031

BMI, body mass index; WC, waist circumference; SBP, systolic blood pressure; DBP, diastolic blood pressure; TC, total cholesterol; TG, triglycerides; LDL, low-density lipoprotein; HDL, high-density lipoprotein; APOA1, apolipoprotein A1; APOB, apolipoprotein B; FPG, fasting plasma glucose; FIN, fasting insulin; HOMA-IR, homeostatic model assessment-insulin resistance; TT, total testosterone; FT, free testosterone; FAI, free androgen index; SHBG, sex hormone-binding globulin; CK, creatine kinase; CKMB, creatine kinase MB; LDH, lactate dehydrogenase; ALT, alanine transferase; AST, aspartate transferase.

### Adjusted Logistic Regression Analysis Between the ApoB/ApoA1 Ratio Quartiles and the Risks of MetS and IR

The associations between the ApoB/ApoA1 ratio and the risk of MetS and IR in PCOS patients are presented in **Table 3**. A significant association between higher ApoB/ApoA1 ratio and increased risk of MetS and IR was found after adjusting for age, BMI, FT, and FAI. Compared with the lowest ApoB/ApoA1 ratio quartile, patients in the highest quartile had a significantly greater OR for both MetS (OR: 24.48, 95%CI: 8.54–70.15, P-trend <0.001) and IR (OR: 1.78, 95%CI: 1.10–2.87, P trend <0.05).

**Table 3:** Adjusted OR (95% CI) for the associations between the ApoB/ApoA1 ratio and the risk of MetS and IR.

Models	Quartile of ApoB/ApoA1 ratio				*P*-_trend_
	Q1 (≤0.45)	Q2 (0.46–0.60)	Q3 (0.61–0.74)	Q4 (>0.74)	
Median	0.39	0.52	0.66	0.86	
MetS, n (%)	4 (1.7%)	14 (5.9%)	54 (22.6%)	118 (49.4%)	
Model 1^a^	1.00 (Reference)	3.69 (1.20–11.38)	16.79 (5.96–47.32)	56.48 (20.30–157.10)	<0.001
P-values		0.023	<0.001	<0.001	
Model 2^b^	1.00 (Reference)	2.29 (0.72–7.35)	7.34 (2.53–21.33)	26.06 (9.16–74.14)	<0.001
P-values		0.162	<0.001	<0.001	
Model 3^c^	1.00 (Reference)	2.14 (0.66–6.90)	7.03 (2.42–20.45)	24.48 (8.54–70.15)	<0.001
P-values		0.173	<0.001	<0.001	
IR, n (%)	55 (22.92%)	79 (33.05%)	119 (49.79%)	148 (61.92%)	
Model 1^a^	1.00 (Reference)	1.62 (1.08–2.45)	3.47 (2.32–5.17)	5.68 (3.77–8.54)	<0.001
P-values		0.02	<0.001	<0.001	
Model 2^b^	1.00 (Reference)	1.14 (0.72–1.79)	1.44 (0.91–2.27)	2.06 (1.29–3.27)	0.001
P-values		0.580	0.116	0.002	
Model 3^c^	1.00 (Reference)	1.12 (0.71–1.77)	1.30 (0.82–2.07)	1.78 (1.10–2.87)	0.012
P-values		0.630	0.261	0.019	

OR, odds ratio; CI, confidence interval; MetS, metabolic syndrome; IR, insulin resistance.

a Model 1 was adjusted by age.

b Based on Model 1, Model 2 was additionally adjusted by body mass index.

c Based on Model 2, Model 3 was additionally adjusted by free testosterone and free androgen index.

Test for trends was based on variables containing the median value for each quartile.

### The Predictive Value of the ApoB/ApoA1 Ratio in Detecting MetS and IR

**Figure 1:**
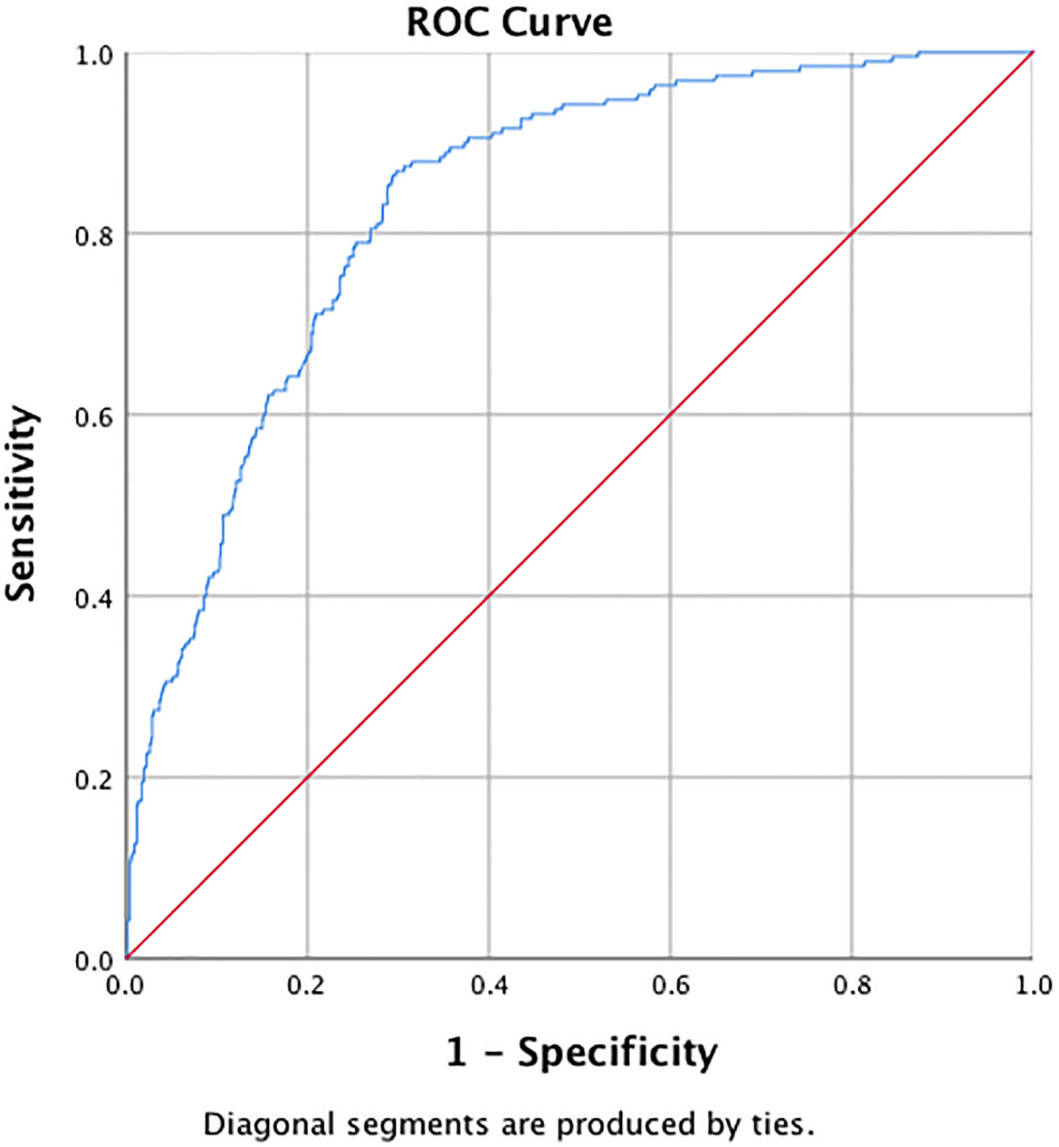
ROC curve for ApoB/ApoA1 ratio to predict MetS in PCOS women.

**Figure 2:**
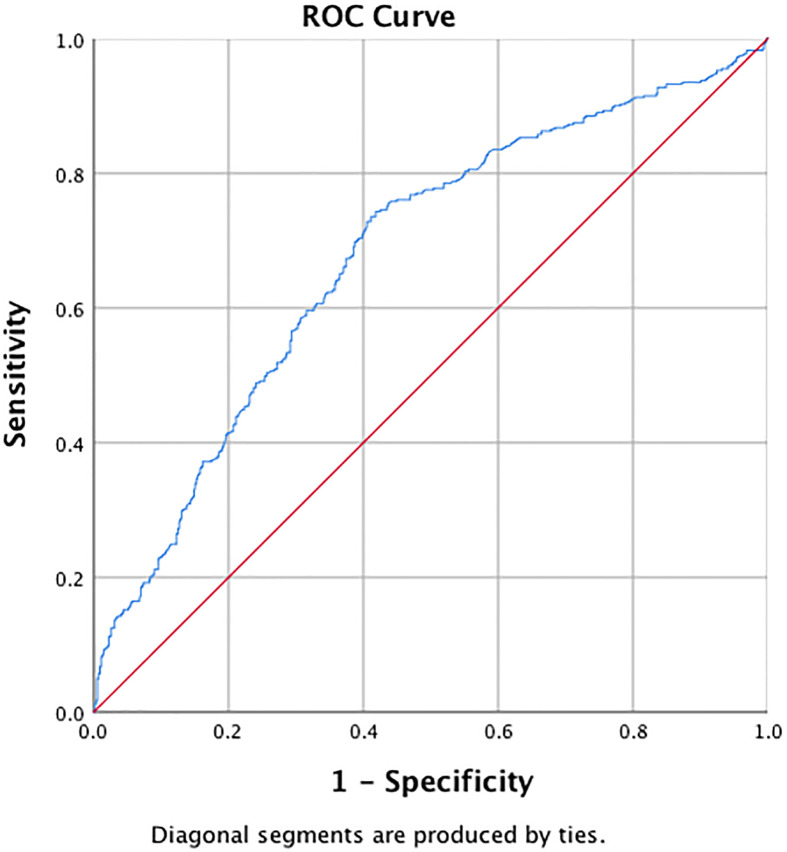
ROC curve for ApoB/ApoA1 ratio to predict IR in PCOS women.

ROC curve analysis of MetS showed that the AUC was 0.84 (95%CI: 0.81–0.86), with a sensitivity of 86.8% and a specificity of 70.3%. The optimal cut-off value of the ApoB/ApoA1 ratio for MetS prediction was 0.64, and the Youden index was 0.57 (**Figure 1**). For IR, the ApoB/ApoA1 ratio had a 74.3% sensitivity and 58.2% specificity with a threshold value of 0.56, the AUC was 0.68 (95%CI: 0.64–0.71), and the Youden index was 0.33 (**Figure 2**).

## Discussion

Our results suggest that an increased ApoB/ApoA1 ratio is associated with worse MetS components, IR, and elevated androgen hormones and liver enzymes and that a higher ApoB/ApoA1 ratio is a promising predictor of MetS and IR in PCOS patients.

MetS, a cluster of dysmetabolic factors, namely, central obesity, hypertension, hyperglycemia, and hyperlipidemia, is prevalent in patients with PCOS and confers increased risk of CVD. Prior studies have suggested that the ApoB/ApoA1 ratio is independently associated with MetS and its components in the general populations among various ethnic groups (13–16). Meanwhile, two studies conducted by Yin et al. (26) and Zheng et al. (27) also reported that PCOS patients with MetS have higher ApoB/ApoA1 ratios than those without MetS. In our study, the ApoB/ApoA1 ratio was significantly correlated with WC, TG, HDL, SBP, and FPG, but not with DBP, and the association was independent of age or obesity. In addition, an increased ApoB/ApoA1 ratio is associated with a higher prevalence of MetS. Our results are in line with the above studies, and furthermore our logistic regression results showed that subjects in the fourth quartile of the ApoB/ApoA1 ratio had a 24.48-fold increased risk of MetS than the first quartile even after adjusting for confounding factors. One study conducted on a Korean population showed that the highest ApoB/A1 ratio quartile resulted in an 8.41-fold increased risk for MetS compared to the lowest quartile (13). Recently, Jing et al. (14) and Chou et al. (16) found that the ApoB/ApoA1 ratio is closely associated with MetS in Chinese populations. Their studies reported 5.18-fold and 3.82-fold increased risks of MetS, respectively, among subjects in the 75th quartile of the ApoB/ApoA1 ratio. Compared with the studies mentioned above, we found a 4-fold greater risk. Taken together, these studies suggest that the ApoB/ApoA1 ratio is a more promising indicator of MetS in the PCOS population than in other populations.

IR has been generally recognized to be the link between MetS and PCOS. Previous studies have suggested that the ApoB/ApoA1 ratio is strongly associated with IR in non-diabetic (17) and type 2 diabetic populations (33) after adjusting for confounding factors. Yin et al. (26) and Zheng et al. (27) also found the ApoB/ApoA1 ratio to be significantly associated with IR in Chinese PCOS patients. In our study, the ApoB/ApoA1 ratio showed a strong positive correlation with FPG, FIN, and HOMA-IR after adjusting for age and BMI, which was in agreement with the above results. Furthermore, our regression analysis suggested that the incidence of IR increased across the ApoB/ApoA1 ratio quartiles. The highest quartile of ApoB/ApoA ratio (OR: 1.78; 95%CI: 1.10–2.87, P-trend <0.05) was independently associated with the presence of IR compared to the lowest quartile after controlling for age, BMI, and androgen hormones. Several possible mechanisms may account for the positive association between the ApoB/ApoA1 ratio and IR. The most widely held hypothesis is that insulin-mediated inhibition of lipase activity is reduced under conditions of IR and that excessive free fatty acids produced by lipolysis then flow into the liver resulting in atherogenic dyslipoproteinemia such as the overproduction of ApoB (34). However, this assumption has certain flaws. Taghibiglou et al. (35) confirmed that overproduction of VLDL-ApoB can lead to an increased activity of protein-tyrosine phosphatase-1B (PTP-1B), which can negatively regulate the insulin signaling pathway thus leading to hepatic IR. In turn, chronic exposure of hepatocytes to high concentrations of insulin appeared to increase PTP-1B, accompanied by a marked suppression of ER-60, a cysteine protease involved in ApoB degradation, thus resulting in an increased synthesis and secretion of ApoB in hepatocytes. In addition, both ApoB and IR are related to an inflammatory state, especially to levels of C-reactive protein, and therefore inflammation might play a potential role in mediating the effect of ApoB on IR. These inconsistencies in the mechanisms of ApoB/ApoA1 in regulating IR need to be further elucidated.

HA is the most significant manifestation of PCOS. HA upregulates the activity of hepatic lipase, which plays an important role in the catabolism of HDL particles leading to reduced HDL (36). We found significant positive associations for TT, FT, and FAI with the ApoB/ApoA1 ratio, but negative associations for SHBG. However, the positive association between the ApoB/ApoA1 ratio and TT disappeared after adjusting for age and BMI, suggesting that FAI, which reflects the biological activity of circulating androgen, is a more sensitive indicator than TT for changes in atherogenic apolipoprotein profiles. Previously, Yin et al. (26) reported a higher ApoB/ApoA1 ratio in obese adolescent PCOS patients with high FAI when compared with non-obese subjects with low FAI, and they concluded that FAI might be involved in obesity-related metabolic changes, which was in accordance with our findings. However, contrasting conclusions were reported by Zheng et al. (27). In their study, the positive associations between FT and FAI and the ApoB/ApoA1 ratio were no longer significant after adjusting for age and BMI together, which indicated that obesity might make more of a contribution to the increased ApoB/ApoA1 ratio than FT and FAI in adult PCOS patients. These inconsistent results might be because HA does not reflect all aspects of atherogenic dyslipoproteinemia, and instead these metabolic disturbances appear to be the combined results of obesity, insulin metabolism, and androgen steroid activity.

Our results also showed positive associations between the ApoB/ApoA1 ratio and ALT and AST regardless of age and obesity, which is consistent with previous reports (24, 25). This relationship can be partly explained by the hepato-ovarian axis (37), and numerous studies have indicated that the characteristics of HA in PCOS patients are linked to NAFLD and to elevated liver enzymes (38–40). In addition, apolipoproteins are mainly produced in hepatocytes, and their production is associated with liver function. ALT and AST are both specific markers of liver damage, thus hepatocellular injury might lead to excessive release of ALT and AST along with overproduction of ApoB. However, we did not find any correlation between the ApoB/ApoA1 ratio with CK, CKMB, or LDH, which is inconsistent with a prior study conducted in myocardial infraction patients (21). Our study nevertheless provides some pieces of evidence for the usefulness of the ApoB/ApoA1 ratio in the early prediction of CVD in PCOS patients because the enrolled patients were much younger and with a low chance of CVD. We suggest that prospective studies should be performed in which CVD-related end points such as myocardial infarction or stroke are studied in the long-term follow-up of PCOS patients in order to further evaluate the predictive value of this ratio.

PCOS tends to be associated with more pronounced metabolic disorders than what is seen in the general population. Concerning the long-term health risks, an understanding of the most sensitive risk indicators for early MetS is of great significance. The literature regarding the ApoB/ApoA1 ratio in MetS is limited, especially regarding PCOS. Our results showed that the AUC_MetS_ was 0.84 and had 86.8% sensitivity and 70.3% specificity with a threshold value of 0.64 and a Youden index of 0.57. In addition, we calculated the ROC curve of the ApoB/ApoA1 ratio for IR in PCOS for the first time, and we took an ApoB/ApoA1 ratio of 0.56 as the cut-off point for IR (with a sensitivity of 74.3% and specificity of 58.2%). Therefore, the association between the ApoB/ApoA1 ratio and obesity, blood pressure, glucose, lipid, and IR parameters confirmed the potential role of the ApoB/ApoA1 ratio in the etiology of metabolic disorders and thus in the occurrence and development of MetS. Traditional lipid indexes including TG, TC, and HDL-C are all risk factors for MetS and CVD. However, their levels vary greatly with dietary fat intake and need at least a 12-hour fast for measurement, which makes this an inconvenient measure for use in clinical practice. In contrast, ApoB and ApoA1 have significant advantages for clinical measurement because their testing is standardized, accurate, automated, low-cost, and does not require fasting by the patient. Therefore, our study supports the use of the ApoB/ApoA1 ratio as a biomarker for MetS and IR in PCOS patients.

The major strengths of the study included the large sample size representing the Chinese PCOS population and the consideration of several potential confounding variables. However, some limitations need to be mentioned. There were only PCOS subjects, and no non-PCOS control group was included. It is of great interest to enroll non-PCOS subjects in order to make comparisons and to further elucidate the role of the ApoB/ApoA1 ratio in metabolic abnormalities. In addition, as a secondary analysis based on the PCOSAct, the ApoB/ApoA1 ratio was only available at baseline, and this cross-sectional study is unable to determine causality and any such association should be further confirmed through longitudinal studies in the future.

## Conclusion

In conclusion, our results showed that an increased ApoB/ApoA1 ratio was associated with worse MetS components, IR, and elevated androgen hormones and liver enzymes. The ApoB/ApoA1 ratio might therefore be a useful tool for screening for MetS and IR among PCOS patients. Larger studies are needed to confirm these findings before they can be applied in the clinic.

## Data Availability Statement

The original contributions presented in the study are included in the article. Further inquiries can be directed to the corresponding author.

## Ethics Statement

The studies involving human participants were reviewed and approved by the First Affiliated Hospital, Heilongjiang University of Chinese Medicine. The patients/participants provided their written informed consent to participate in this study.

## Author Contributions

HH, JF, and SZ performed most of the work for the study, wrote the manuscript, and prepared the final version of the manuscript. XW designed and conceived the study. JL took part in the study design and helped in the revision of the manuscript. YW, JC, JG, and YG helped with data collection, statistical analysis, and interpretation. All authors contributed to the article and approved the submitted version.

## Funding

1. National Key R&D Program of China, Research and Development for Modernization of Chinese Medicine, Evidence-based Evaluation of the Program of Integration of Traditional Chinese and Western Medicine for High Incidence of Gynecological Diseases (No.2019YFC1709500); 2. National Clinical Cooperation Pilot Program of TCM and Western Medicine for Major and Difficult Diseases (National Office of Traditional Chinese Medicine, No.[2018]3), Combined Traditional Chinese and Western Medicine Infertility and Assisted Reproductive Technology; 3. Scientific Research Fund of Heilongjiang University of Chinese Medicine, Study on Acupuncture Combined with Traditional Chinese Medicine Compound to Improve the Live Birth Rate of Patients with *In Vitro* Fertilization and Embryo Transfer (No. 2019BS09).

## Conflict of Interest

The authors declare that the research was conducted in the absence of any commercial or financial relationships that could be construed as a potential conflict of interest.

## Publisher’s Note

All claims expressed in this article are solely those of the authors and do not necessarily represent those of their affiliated organizations, or those of the publisher, the editors and the reviewers. Any product that may be evaluated in this article, or claim that may be made by its manufacturer, is not guaranteed or endorsed by the publisher.
